# Sodium fluorocitrate having inhibitory effect on fatty acid uptake ameliorates high fat diet-induced non-alcoholic fatty liver disease in C57BL/6J mice

**DOI:** 10.1038/s41598-019-54476-5

**Published:** 2019-11-28

**Authors:** Seung A. Hong, Ik-Rak Jung, Sung-E. Choi, Yoonjung Hwang, Soo-Jin Lee, Youngho Son, Yu Jung Heo, Rihua Cui, Seung Jin Han, Hae Jin Kim, Kwan Woo Lee, Yup Kang

**Affiliations:** 1 Department of Physiology, Ajou University School of Medicine, Suwon, Gyunggi-do 443-749 Republic of Korea; 2 Department of Biomedical Science, The Graduate School, Ajou University, Suwon, Gyunggi-do 443-749 Republic of Korea; 3 Department of Endocrinology and Metabolism, Ajou University School of Medicine, Suwon, Gyunggi-do 443-749 Republic of Korea

**Keywords:** Non-alcoholic steatohepatitis, Non-alcoholic fatty liver disease

## Abstract

Non-alcoholic fatty liver disease (NAFLD) is excessive fat build-up in the liver without alcohol consumption and includes hepatic inflammation and damage. Excessive influx of fatty acids to liver from circulation is thought to be a pathogenic cause for the development of NAFLD. Thus, inhibition of fatty acid intake into hepatocyte would be a maneuver for protection from high fat diet (HFD)-induced NAFLD. This study was initiated to determine whether sodium fluorocitrate (SFC) as a fatty acid uptake inhibitor could prevent palmitate-induced lipotoxicity in hepatocytes and protect the mice from HFD-induced NAFLD. SFC significantly inhibited the cellular uptake of palmitate in HepG2 hepatocytes, and thus prevented palmitate-induced fat accumulation and death in these cells. Single treatment with SFC reduced fasting-induced hepatic steatosis in C57BL/6J mice. Concurrent treatment with SFC for 15 weeks in HFD-fed C57BL/6J mice prevented HFD-induced fat accumulation and stress/inflammatory signal activation in the liver. SFC restored HFD-induced increased levels of serum alanine aminotransferase and aspartate aminotransferases as hepatic injury markers in these mice. SFC treatment also improved HFD-induced hepatic insulin resistance, and thus ameliorated HFD-induced hyperglycemia. In conclusion, inhibition of fatty acid mobilization into liver through SFC treatment can be a strategy to protect from HFD-induced NAFLD.

## Introduction

Non-alcoholic fatty liver disease (NAFLD) is a liver disease that occurs when fat is deposited in the liver due to causes other than excessive alcohol use and represents the most common cause of chronic liver disease. NAFLD affects up to 25% of the global population and is thus becoming a serious global health concern^1,2^. NAFLD has a spectrum ranging from simple steatosis to non-alcoholic steatohepatitis (NASH), progressing to cirrhosis^3^. Steatosis is defined by the presence of lipid within the cytoplasm of hepatocytes, the criterion for which is determined as being hepatic lipid level greater than 5% of the liver weight^4^. On the other hand, NASH is defined as steatosis in the presence of hepatic inflammation and injury. NASH frequently includes hepatic fibrosis, replacing the tissue with type 1 collagen^5,6^. NAFLD is also regarded as the hepatic manifestation of the metabolic syndrome, and is thus associated with obesity, diabetes and cardiovascular disease^7,8^.

Hepatic steatosis is due to an imbalance between the synthesis and removal of lipids in liver. It is regarded as a common complication of obesity^9,10^. Multiple metabolic pathways contribute to the fat accumulation in hepatocytes, including high influx of fatty acids, excessive *de novo* lipogenesis (DNL), decreased fatty acid oxidation, and reduced secretion of very low density lipoprotein (VLDL) in the liver^11,12^. In a postprandial state, chylomicron transports dietary fats into systemic circulation, where the fats can be delivered to the liver through hepatic uptake of fatty acids^13,14^. In particular, overload of lipid diet can cause fatty acid spillover through lipoprotein lipase-mediated chylomicron hydrolysis in adipose tissues and easily lead to hepatic steatosis through enhanced mobilization of fatty acid into liver^15,16^. On the other hand, large quantity of free fatty acids are also released into circulation from adipose tissues through activation of hormone-sensitive lipase under long-term fasting and insulin resistance conditions and delivered to the liver tissues^17,18^. If delivered fatty acid exceeds the demand for lipid oxidation in liver, surplus fatty acids can be re-esterified to triacylglycerol within hepatocytes. In high fat diet-fed condition, continuous supply of dietary fat exceeding the storage capacity of adipose tissue may induce insulin resistance, resulting in hepatic steatosis through augmented hydrolysis of lipid in adipose tissues and enhanced mobilization of fatty acids into hepatocytes. In humans having NAFLD, approximately 60% of hepatic triacylglycerol have been reported to originate from fatty acids released from white adipose tissues^19^. Continuous feeding of high fat diet (HFD) in C57BL/6J mice has been widely used as an animal model for the development of NAFLD^20^.

The mechanism of progression of simple steatosis to steatohepatitis is not completely understood yet. Although early studies have suggested that fat accumulation in the liver is essential for the development of NASH, steatosis is not thought to be an essential prerequisite for the NASH development^21,22^. Rather than accumulated fat itself, dysregulation of lipid homeostasis caused by an increased influx or impaired oxidation of free fatty acids has been suggested to play a role in the induction of NASH development^23^. In particular, accumulation of toxic lipid intermediates such as phosphatidic acid, lysophosphatidic acid, lysophosphatidyl choline, ceramide, and diacylglycerol metabolized from fatty acids has been reported to contribute to hepatocellular injury^3,24,25^. On the other hand, it was also reported that saturated fatty acids such as palmitate and stearic acid are known to be toxic to hepatocytes whereas unsaturated fatty acids are not and even protective against saturated fatty acid-induced lipotoxicity^26^. Therefore, development of NASH has been viewed as a consequence of saturated fatty acid-induced lipotoxicity to hepatocytes^25^. Lipotoxic species can affect the hepatic cell behavior via multiple mechanisms, including induction of inflammatory pathway through inflammasome and toll-like receptor (TLR), endoplasmic reticulum stress responses, and oxidative stress responses through mitochondrial dysfunction, and activation of death signals^27,28^. Increased levels of phospho-form of C-Jun N-terminal kinase (P-JNK) and nuclear factor kappa B (NFκB) representing signal activation of cellular stress and inflammation have been reported to be typical mediators for the induction of lipotoxicity in NASH^29^. Phospho-AKT insulin signaling pathway as an indicator for insulin sensitivity and cell survival is also down-regulated in the liver of HFD-induced NASH^30^.

Sodium fluorocitrate (SFC) is a metabolic derivative converted from sodium fluoroacetate (SFA), which was originally used for the eradication of mammalian pests^31^. SFC is known to bind to tricarboxylic acid (TCA) cycle enzyme aconitase and inhibit its activity, thereby halting the TCA cycles. Thus, many features of SFA poisoning were supposed to be direct or indirect consequences of impaired oxidative metabolism and energy depletion through the inhibition of aconitase^32^. On the other hand, a recent study demonstrated that low dose of SFC was specifically protective against palmitate-induced lipotoxicity in INS-1 beta cells, and its protective activity was due to its inhibitory activity against fatty acid uptake into beta cells, rather than inhibitory activity against aconitase^33^.

To determine whether liver is sensitive to the inhibitory effect of SFC on fatty acid uptake, BODIPY-palmitate in conjunction with SFC was intravenously injected into C57BL/6J mice, and the reducing effect of SFC on BODIPY fluorescence in liver tissues was then investigated. Our studies were also initiated to determine whether SFC could prevent palmitate-induced lipotoxicity in HepG2 hepatocytes and protect C57BL/6J mice from HFD-induced NAFLD. The preventive effect of SFC on palmitate-induced toxicity was determined by investigating the inhibitory effect of SFC on palmitate-induced cell death and stress/inflammatory signal activation. On the other hand, the protective effect of SFC on HFD-induced NAFLD was determined by investigating the inhibitory effect of SFC on HFD-induced fat accumulation, macrophage infiltration, stress/inflammatory signal activation, inflammatory gene expression, and hepatic injury in HFD-fed C57BL/6J mouse liver. The preventive effect of SFC against HFD-induced insulin resistance and hyperglycemia was also investigated by measuring glucose levels in glucose tolerance test (GTT), insulin tolerance test (ITT), and pyruvate tolerance test (PTT).

## Results

### SFC inhibits fatty acid uptake into liver tissues

**Figure 1:**
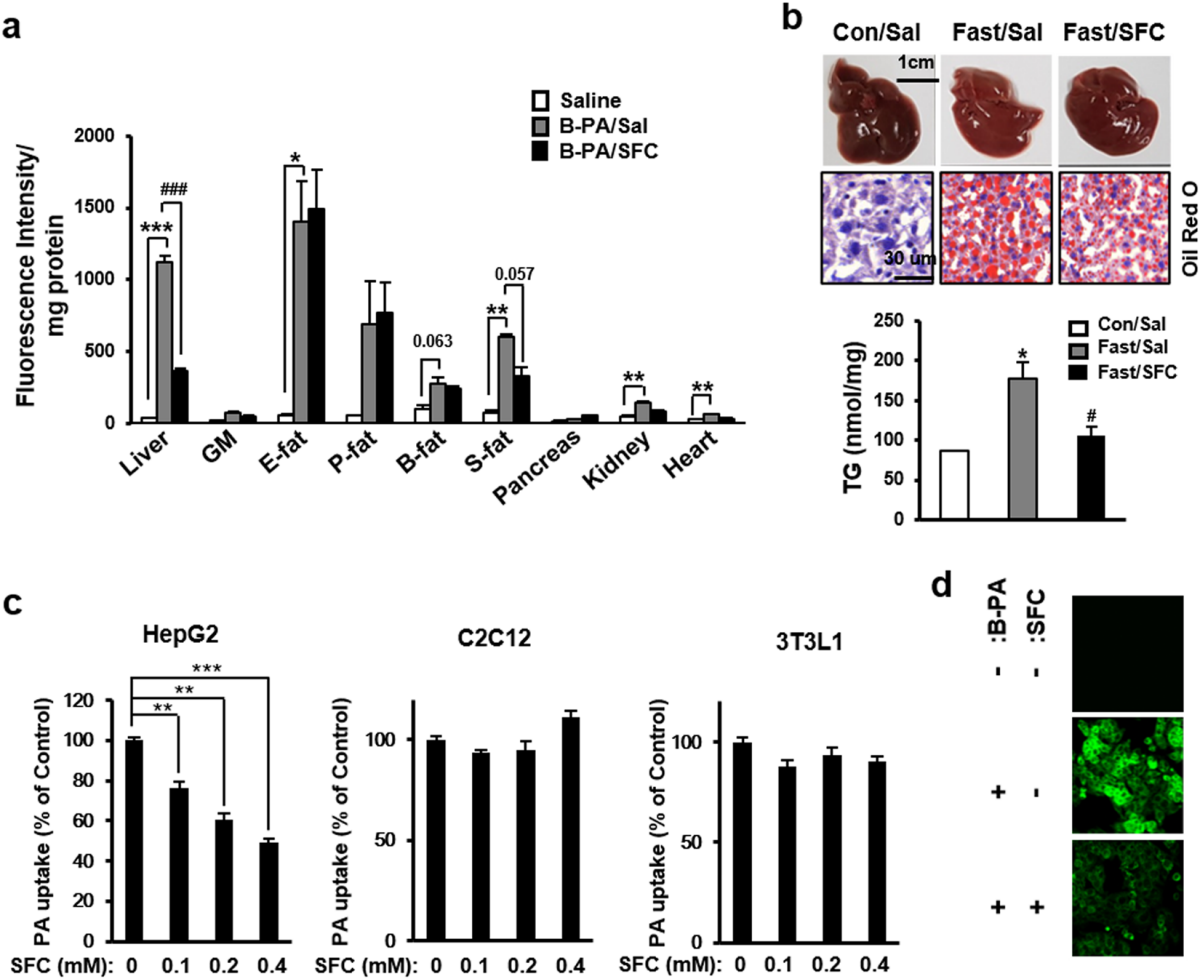
Sodium fluorocitrate (SFC) inhibits fatty acid uptake in hepatocytes. (**a**) Uptake of BODIPY-palmitate (B-PA) in different tissues (GM: gastrocnemius muscle, E-fat: epididymal fat, P-fat, perirenal fat, B-fat: brown fat, S-fat: subcutaneous fat) was determined by measuring the fluorescence intensity of tissue extract isolated from B-PA (50 μg/mouse)-injected C57BL6J mice after SFC treatment (20 mg/kg). **p < 0.01; ***p < 0.001 vs. saline-treated mice (Sal). ^###^*p* < 0.001 vs. B-PA and saline-treated mice (B-PA/Sal). (**b**) A total of twelve 8-week-old male mice were assigned to three groups: Con/Sal (n = 4); Fast/Sal (n = 4); and Fast/SFC (n = 4). Mice were intraperitoneally injected with saline (Sal) or SFC (20 mg/kg) and then fasted for 15 h (Fast). Representative livers of non-fasted (Con/Sal) and fasted mice with (Fast/SFC) or without SFC treatment (Fast/Sal) are shown. Liver sections were stained with Oil-Red O and the nuclei were then counterstained with hematoxylin. Triacylglycerol (TG) content per 1 mg of wet liver tissues was measured by using TG assay kit. *p < 0.05 vs. control non-fasted mice (Con/Sal). ^#^*p* < 0.05 vs. saline-treated 15 h-fasted mice ^(^Fast/Sal). (**c**) Cellular uptake of palmitate (PA) was determined by measuring the fluorescence of B-PA in HepG2 hepatocytes, C2C12 myocytes or 3T3L1 adipocytes after incubation with 2.0 μM B-PA for 3 h in the presence of different concentration of SFC. **p < 0.01; ***p < 0.001 vs. B-PA-treated cells. (**d**) Florescent images of B-PA-treated HepG2 cells with or without 0.2 mM SFC treatment were visualized by Zeiss 710 confocal microscopy (489 nm as excitation and 510 nm as emission).

A previous report demonstrated that SFC has an inhibitory effect on fatty acid uptake in beta cells^33^. To determine which tissues are sensitive to the inhibitory effect of SFC on fatty acid uptake, BODIPY-palmitate was intravenously injected into SFC-treated C57BL/6J mice, and SFC’s preventive effect on BODIPY fluorescence intake in different tissues was then investigated. Figure 1a shows that liver and fat tissues revealed a relatively high intensity of fluorescence, suggesting that these tissues have high capacity for fatty acid uptake. Interestingly, SFC significantly reduced BODIPY-induced fluorescence in liver tissues, suggesting that liver tissue is sensitive to SFC’s inhibitory effect on fatty acid uptake. On the other hand, muscle, pancreas, kidney, and heart tissues showed low intensity of fluorescence, and SFC’s inhibitory effect on fatty acid uptake was not significant in these tissues. Since liver tissue was sensitive to SFC’s inhibitory effect on fatty acid uptake and an early study had reported that long-term fasting induced enhanced mobilization of fatty acid to liver from adipose tissues^34^, it was determined whether SFC could reduce lipid accumulation in fasted mouse liver. Figure 1b shows that fasting for 15 h in C57BL/6J mice increased lipid accumulation in liver. Liver color was light brown, and Oil Red O staining showed the existence of red oil droplets in the fasting mouse liver. SFC treatment in fasting mice thickened the brown color in liver and reduced the size of red lipid droplets in the liver tissues (Fig. 1b). Liver triacylglycerol level in fasting mice was increased by 106%, compared to that in *ab libitum* feeding mice. However, SFC treatment in fasting mice decreased the triacylglycerol level by approximately 40%, compared to that of SFC-untreated mice.

These data suggest that SFC treatment could reduce fasting-induced hepatic steatosis, possibly through the inhibition of fatty acid uptake into hepatocytes. SFC’s inhibitory effect on fatty acid uptake was also investigated in cultured hepatocytes. Figure 1c shows that SFC prevented fatty acid uptake into HepG2 hepatocytes in a concentration-dependent manner. Treatment with 0.4 mM SFC reduced fluorescence intensity by BODIPY-palmitate to 49% of intensity in SFC-untreated cells. On the other hand, SFC did not show significant preventive effect on fatty acid uptake in differentiated C2C12 muscle cells and 3T3 L1 adipose cells (Fig. 1c). In addition, green fluorescence image was observed in BODIPY-palmitate-treated HepG2 cells, and SFC reduced the intensity of fluorescence (Fig. 1d). Conclusively, all these data support that SFC has a selective inhibitory effect on fatty acid uptake in hepatocytes.

### SFC prevents palmitate-induced lipotoxicity in HepG2 hepatocytes

**Figure 2:**
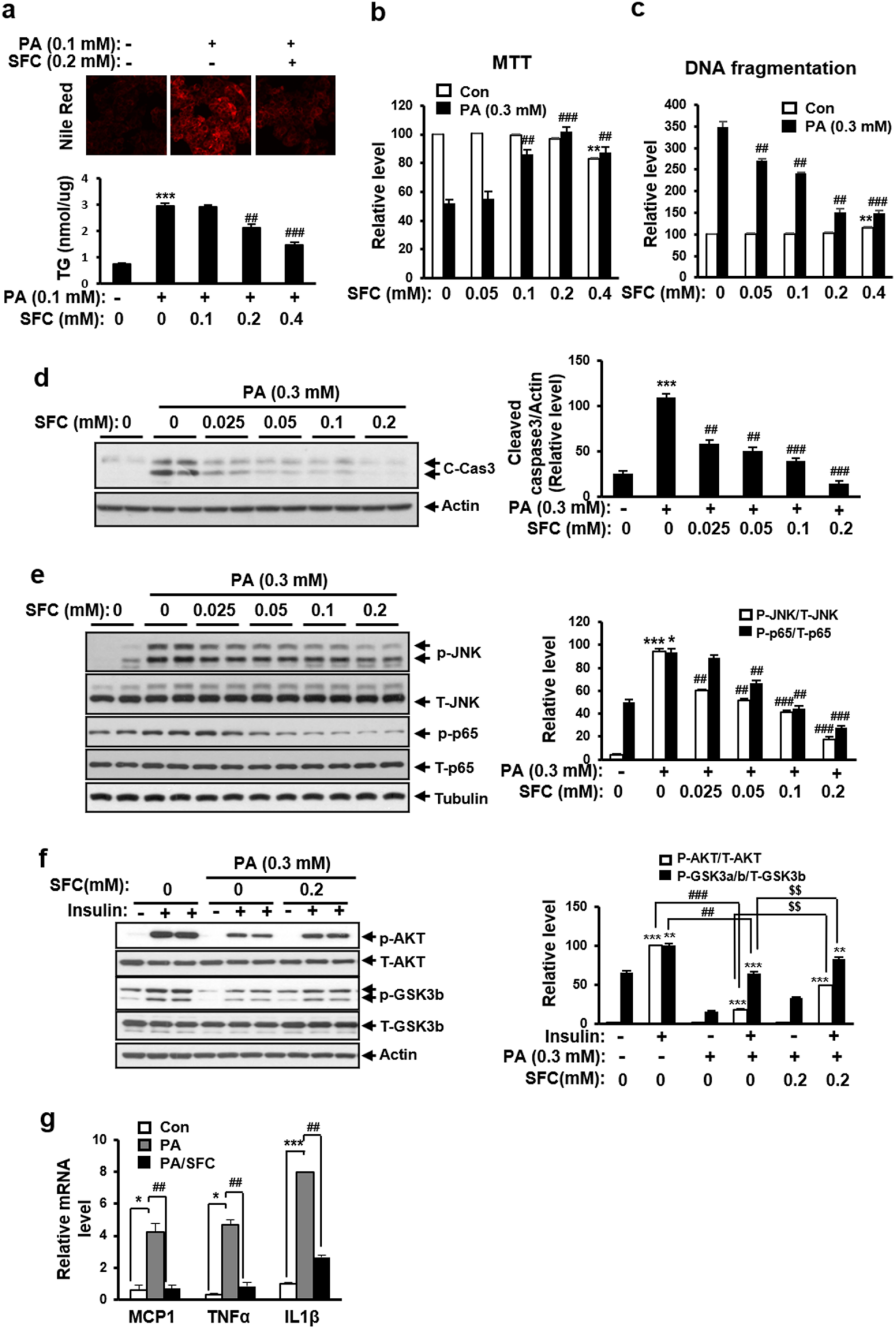
SFC reduces palmitate (PA)-induced lipotoxicity in HepG2 hepatocytes. (**a**) SFC reduces PA-induced lipid accumulation in HepG2 cells. HepG2 cells were exposed on 0.1 mM PA for 24 hrs in the presence or absence of 0.2 mM SFC, and the intracellular lipid was then observed by Nile Red staining (540 as excitation and 630 as emission). TG per 1 μg of cellular protein was quantified by using TG assay kit. ***p < 0.001 vs. PA-untreated cells. ^###^*p* < 0.001 vs. PA-treated cells. (**b**) Viability reduction by PA treatment (0.3 mM, 15 h) in HepG2 cells and protective effect of SFC on the viability reduction was determined by MTT viability assay. **p < 0.01 vs. PA-untreated cells. ^##^*p* < 0.01; ^###^*p* < 0.001 vs. PA-treated cells. (**c**) Cell death by PA treatment (0.3 mM, 15 hrs) in HepG2 cells and protective effect of SFC on cell death was determined by Cell Death Detection enzyme-linked immunosorbent assay. **p < 0.01 vs. PA-untreated cells. ^##^*p* < 0.01; ^###^*p* < 0.001 vs. PA-treated cells. (**d**) Cell death was determined by measuring level of cleaved caspase 3 in immunoblotting. ***p < 0.001 vs. PA-untreated cells. ^##^*p* < 0.01; ^###^*p* < 0.001 vs. PA-treated cells. Full-length blots are included in Supplementary Fig. 4 of Supplementary Information (SI). (**e**) Stress and inflammatory signal activation by PA treatment (0.3 mM, 12 h) and protective effect of SFC on the activation were determined by measuring levels of phospho-N-terminal C-JUN kinase (p-JNK) and phospho-P65 subunit of NFkB (p-p65) in immunoblotting. **p < 0.01 vs. PA-untreated cells. ^##^*p* < 0.01; ^###^*p* < 0.001 vs. PA-treated cells. Full-length blots are included in Supplementary Fig. 5 of SI. (**f**) Insulin resistance by PA treatment and protective effect of SFC on the insulin resistance were determined by measuring levels of phospho-protein kinase B (p-AKT) and phospho-glycogen synthase kinase 3b (p-GSK3b) in immunoblotting after 30 min insulin stimulation. **p < 0.01; ***p < 0.01 vs. insulin-unstimulated cells. ^##^*p* < 0.01; ^###^*p* < 0.001 vs. insulin-stimulated and PA-untreated cells. ^$$^*p* < 0.01 vs. insulin-stimulated and PA-treated cells. Full-length blots are included in Supplementary Fig. 6. (**g**) Inflammatory gene induction by PA treatment and preventive effect of SFC on the induction were determined by real-time PCR. *p < 0.05; ***p < 0.001 vs. PA-untreated cells. ^##^*p* < 0.01 vs. PA-treated cells.

Since SFC has an inhibitory effect on fatty acid uptake in hepatocytes, it was determined whether SFC treatment was protective against palmitate-induced fat accumulation and lipotoxicity in HepG2 hepatocytes. Initially, fat accumulation was induced by long-term treatment with non-toxic low concentration of palmitate in HepG2 cells. The palmitate-induced fat accumulation was investigated by Nile Red staining of neutral lipid in cells. Figure 2a shows that red fluorescence demonstrating the accumulation of lipid was detected in HepG2 cells treated with 0.1 mM palmitate for 24 h. Concurrent treatment with 0.2 mM SFC fairly reduced the intensity of red fluorescence. In accordance with the reduction of red fluorescence, SFC prevented palmitate-induced triacylglycerol increase in an SFC concentration-dependent manner (Fig. 2a). Treatment with 0.4 mM SFC reduced the triacylglycerol level in palmitate-treated HepG2 cells by approximately 50%, compared with palmitate-treated cells without SFC. Next, the preventive effect of SFC on palmitate-induced lipotoxicity was investigated by the restoration of palmitate-induced viability reduction, DNA fragmentation, and caspase 3 cleavage. Figure 2b,c show that treatment with 0.3 mM palmitate for 15 h reduced cell viability but increased DNA fragmentation to 52% and 348%, respectively. SFC treatment prevented palmitate-induced viability reduction and DNA fragmentation in an SFC concentration-dependent manner. Treatment with 0.2 mM SFC restored palmitate-induced viability reduction nearly completely and reduced palmitate-induced DNA fragmentation by approximately 57%. Palmitate also increased the level of cleaved form of caspase 3, and SFC inhibited the palmitate-induced cleavage of caspase 3 in an SFC concentration-dependent manner (Fig. 2d). Palmitate increased stress/inflammatory signals as mediators for lipotoxicity to HepG2 cells. Phospho-form of C-Jun N-terminal kinase (JNK) as a mitogen-activated kinase activated by oxidative, endoplasmic reticulum, or inflammatory stress was increased by palmitate treatment. Phospho-form of NFkB subunit p65 as a signal mediator for inflammation was also increased by palmitate treatment. However, SFC treatment in conjunction with palmitate reduced levels of phospho-JNK and phospho-p65 in an SFC concentration dependent manner (Fig. 2e). In accord with inflammatory signal activation, the expression levels of inflammatory genes, such as MCP-1, TNFα and IL-1β were increased by palmitate treatment, and SFC significantly prevented the palmitate-induced upregulation of inflammatory gene expression (Fig. 2g). In addition, insulin signaling molecules as markers of insulin sensitivity and cell survival were investigated by measuring levels of phospho-AKT and phospho-GSK3b through immunoblotting. Figure 2f shows that levels of insulin-stimulated phospho-AKT and phospho-GSK3b were reduced by palmitate treatment, and that SFC significantly restored the palmitate-induced decrease of insulin-stimulated signals.

### SFC improves high fat diet-induced steatosis in C57BL/6J mice

**Figure 3:**
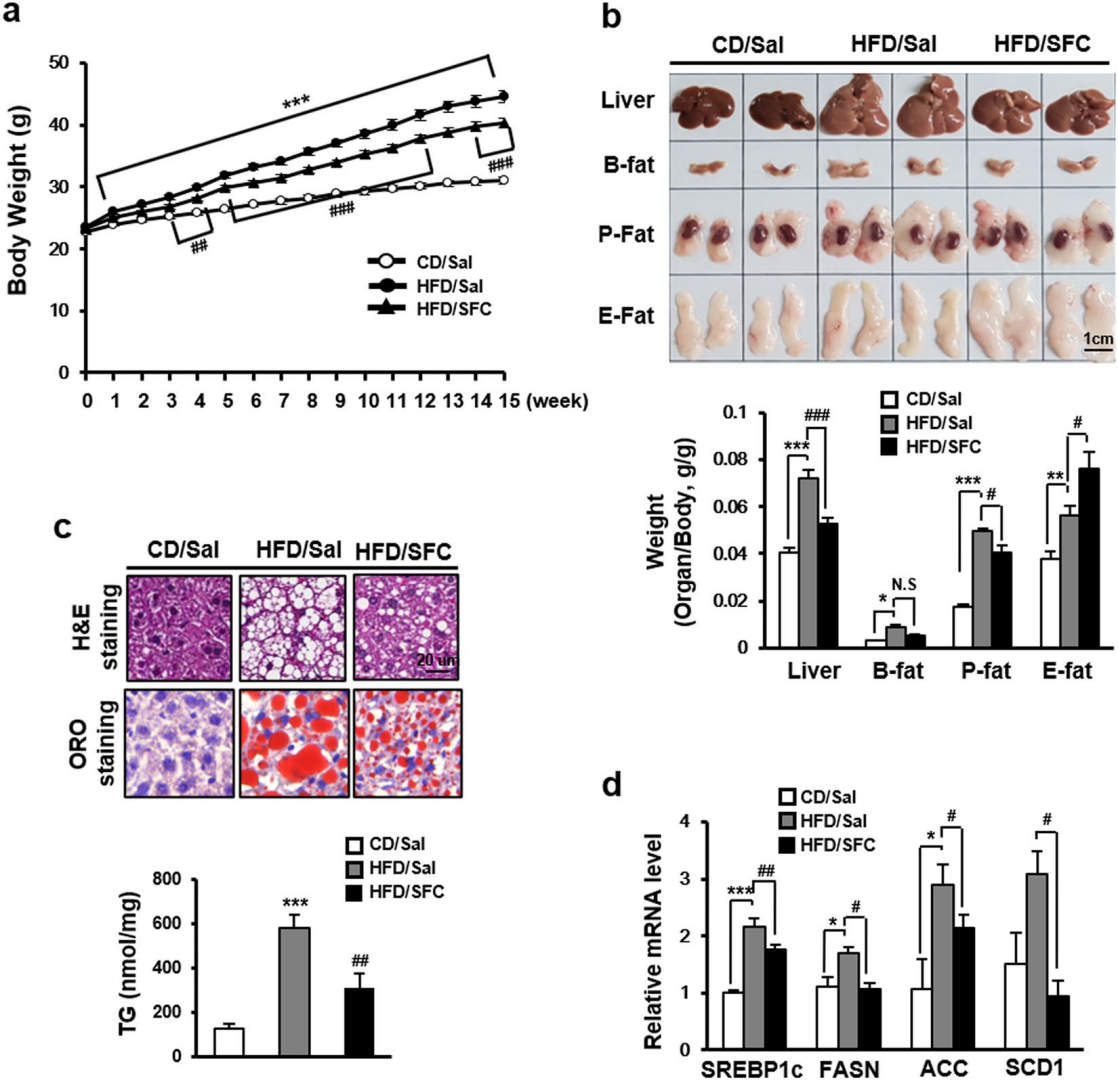
SFC reduces high fat diet (HFD)-induced steatosis in C57BL/6J mice. (**a**) Body weight of HFD-fed mice with or without SFC treatment (10 mg/kg, every other day, for 15 weeks) was measured every week. ***p < 0.01 vs. Saline-treated chow diet (CD)-fed mice. ^##^*p* < 0.01; ^###^*p* < 0.001 vs. saline-treated high fat diet (HFD)-fed mice. (**b**) Representative pictures and average weight ratios of livers, brown fats (B-fat), perirenal fats (P-fat), and epididymal fats (E-fat) isolated from HFD-fed mice with or without SFC treatment are shown. Weight ratios of organs were determined by dividing organ weight with mouse weight individually. *p < 0.05; **p < 0.01; ***p < 0.001 vs. CD mice. ^#^*p* < 0.01; ^###^*p* < 0.001 vs. HFD mice. (**c**) Liver sections stained with Hematoxylin & Eosin or Oil-Red O isolated from HFD with or without SFC treatment. TG content per 1 mg of liver tissues was measured by using TG assay kit. ***p < 0.001 vs. CD mice. ^#^*p* < 0.05 vs. HFD mice. (**d**) RNA levels in livers isolated from HFD-fed mice with or without SFC treatment were determined by real-time PCR. *p < 0.05; ***p < 0.001 vs. CD mice. ^#^*p* < 0.05; ^##^*p* < 0.001 vs. HFD mice.

Since enhanced fatty acid intake to hepatocytes can be a pathogenic cause for hepatic steatosis and SFC has an inhibitory effect on fatty acid uptake into hepatocyte, it was determined whether SFC could prevent hepatic steatosis in HFD-fed mice. High fat diet (HFD) was *ad libitum* fed to C57BL/6J mice for 15 weeks, starting at 8 weeks of age and SFC (10 mg/kg) was intraperitoneally injected every other day for the same 15 weeks. Figure 3a shows that mice fed by HFD were heavier than control chow diet (CD)-fed mice at all ages from the 1^st^ week of experiment. SFC treatment slightly reduced the weight of HFD-fed mice at most ages. The amount of food intake in HFD group was lower than in CD group, but not different from that in the SFC-treated HFD-fed group (HFD/SFC) (Supplementary Fig. 1 of the Supplementary Information (SI)). In accordance with weight increase, the weights of liver and fat were significantly increased in HFD group compared with in CD group (Fig. 3b). However, SFC treatment significantly prevented the HFD-induced increase of liver weight. Interestingly, SFC did not reduce the weight of white adipose tissues in HFD-fed mice and, even increased the weight of epididymal fat (Fig. 3b). Triacylglycerol amount in liver was increased by approximately 350% in HFD group compared with that in CD group, and SFC treatment significantly prevented the increase of liver triacylglycerol by HFD (Fig. 3c). Hematoxylin and Eosin (H&E) staining showed existence of lipid droplets and Oil Red O (ORO) staining clearly demonstrated red-colored lipid droplets with larger size in liver tissues of HFD group (Fig. 3c). SFC treatment reduced the number and size of lipid droplets in liver tissues of HFD group (Fig. 3c). In accordance with the accumulation of lipid in liver of HFD-fed mice, the expression of genes involved in lipid synthesis, such as sterol responsive element binding protein 1c (SREBP1c), fatty acid synthase (FASN), acetyl-CoA carboxylase (ACC), and stearyl CoA desaturase (SCD) was increased in liver of HFD group. SFC treatment significantly prevented the increase of expression in HFD-fed mice (Fig. 3d).

### SFC prevents high fat-induced hepatic inflammation in C57BL/6J mice

**Figure 4:**
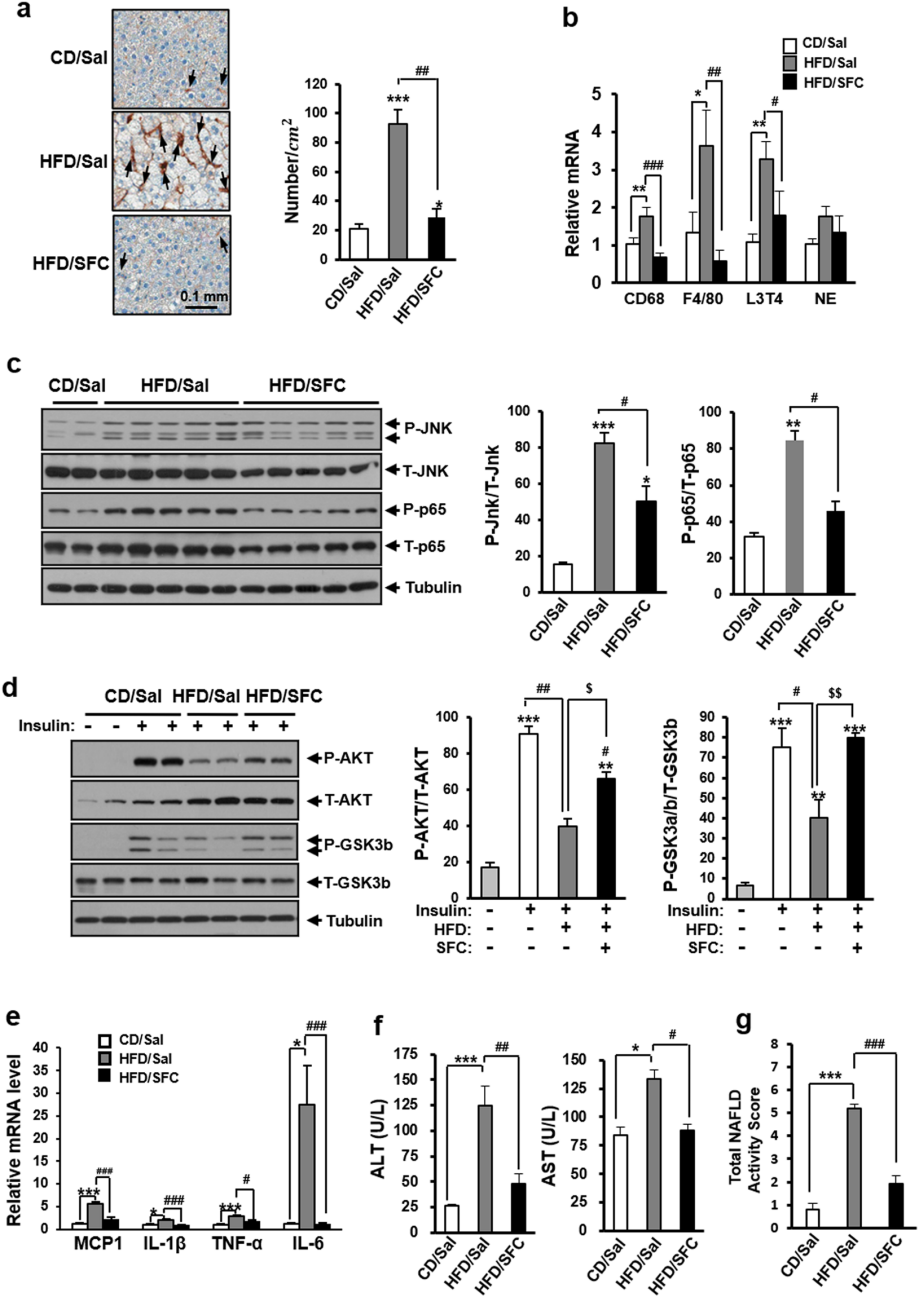
SFC reduces HFD-induced steatohepatitis in C57BL/6J mice. (**a**) Macrophages in liver tissues were stained with anti-F4/80 antibodies, and then counterstained with Hematoxylin. Arrows designate stained macrophages. Numbers of brown cells with small nucleus were counted in 1 cm × 1 cm of tissue. (**b**) Relative expression of genes expressed in immunocytes (CD68: macrophage glycoprotein, F/4/80: macrophage protein, L3T4: T lymphocyte glycoprotein, NE: neutrophil elastase) was determined by real-time PCR. *p < 0.05; **p < 0.01; ***p < 0.001 vs. CD mice. ^#^*p* < 0.05; ^##^*p* < 0.01; ^###^*p* < 0.001 vs^.^ HFD mice. (**c)** Levels of signaling molecules related to stress/inflammation were analyzed by immunoblotting with anti-P-JNK and anti-P-p65 antibodies. Two representative bands from CD/Sal (n = 4) and all five bands from HFD/Sal (n = 5) or HFD/SFC (n = 5) groups were shown in immunoblotting images. *p < 0.05; **p < 0.01; ***p < 0.001 vs. CD mice. ^#^*p* < 0.05 vs. HFD mice. Full-length blots are included in Supplementary Fig. 7 of SI. (**d**) Levels of insulin signaling molecules after insulin stimulation were analyzed by immunoblotting with anti-AKT and anti-GSK3b antibodies. Two representative bands from each group (n = 4) are shown in immunoblotting images. **p < 0.01; ***p < 0.001 vs. insulin-unstimulated CD mice. ^#^*p* < 0.05; ^##^*p* < 0.01 vs. insulin-stimulated CD mice. ^$^*p* < 0.05; ^$$^*p* < 0.01 insulin-stimulated HFD mice. Full-length blots are included in Supplementary Fig. 8 od SI. (**e**) Relative expression of genes related to inflammation was determined by real-time PCR. *p < 0.05; ***p < 0.001 vs. CD mice. ^#^*p* < 0.05; ^###^*p* < 0.001 vs. HFD mice. (**f**) Hepatic injury was determined by measuring the levels of alanine transaminase (ALT) and aspartate transaminase (AST) in plasma by using an autochemical analyzer^.^ *p < 0.05; ***p < 0.001 vs. CD mice. ^#^*p* < 0.05; ^##^*p* < 0.01 vs. HFD mice.

To determine whether SFC reduces steatohepatitis in HFD-fed C57BL/6J mice, recruitment of macrophage in liver tissues was initially investigated by F4/80 immunostaining. Figure 4a shows that HFD for 15 weeks increased the number of F4/80-positive cells in liver tissues by 344% compared with CD group, and SFC treatment reduced the number of F4/80-positive cells in HFD-fed mouse liver close to that of CD group. In addition, SFC reduced the expression level of CD68 and F4/80 as macrophage genes and L3T4 as a T lymphocyte gene in HFD-fed mouse liver (Fig. 4b). However, SFC did not reduce the expression level of neutrophil elastase gene (NE) as neutrophil marker. These data suggest that SFC treatment prevents the HFD-induced infiltration of immunocytes including macrophage. In accordance with SFC’s inhibitory effect on HFD-induced macrophage infiltration, SFC treatment prevented HFD-induced stress/inflammatory signal activation in liver tissues. Figure 4c shows that SFC treatment significantly reduced the levels of phospho-JNK and phospho-p65 as stress/inflammatory signals in HFD-fed mouse livers. In accordance with SFC’s preventive effect against HFD-induced inflammatory signals, SFC significantly reduced the HFD-induced expression of inflammatory genes, such as MCP1, IL-1β, TNF-α and IL-6 in liver tissue (Fig. 4e). On the other hand, HFD reduced the levels of insulin-stimulated phospho-AKT and phospho-GSK3b in liver tissues, and SFC restored the reduced levels of insulin-stimulated phospho-AKT and phospho-GSK3b in HFD group (Fig. 4d). The preventive effect of SFC against hepatic damage in HDF-fed mouse liver was also observed. SFC significantly reduced the levels of alanine aminotransferase (ALT) and aspartate aminotransferase (AST) as hepatic damage markers in HFD-fed mouse plasma (Fig. 4f). On the other hand, NAFLD activity score (NAS) defined as sum of the scores for steatosis (0–3), inflammation (0–3), and ballooning degeneration (0–2) was determined as a clinical diagnosis of NAFLD. HFD increased NAS to 5.2, representing a histologic diagnosis of NASH. SFC significantly reduced HFD-induced NAS to 1.93, demonstrating that SFC could prevent HFD-induced NASH (Fig. 4g, Supplementation Table 1 of Supplementary Information (SI)). All these data suggest that HFD for 15 weeks induces hepatic inflammation and injury in C57BL/6J mice, and that SFC treatment prevents the HFD-induced hepatic inflammation and injury.

### SFC improves high fat-induced hyperglycemia in C57BL/6J mice

**Figure 5:**
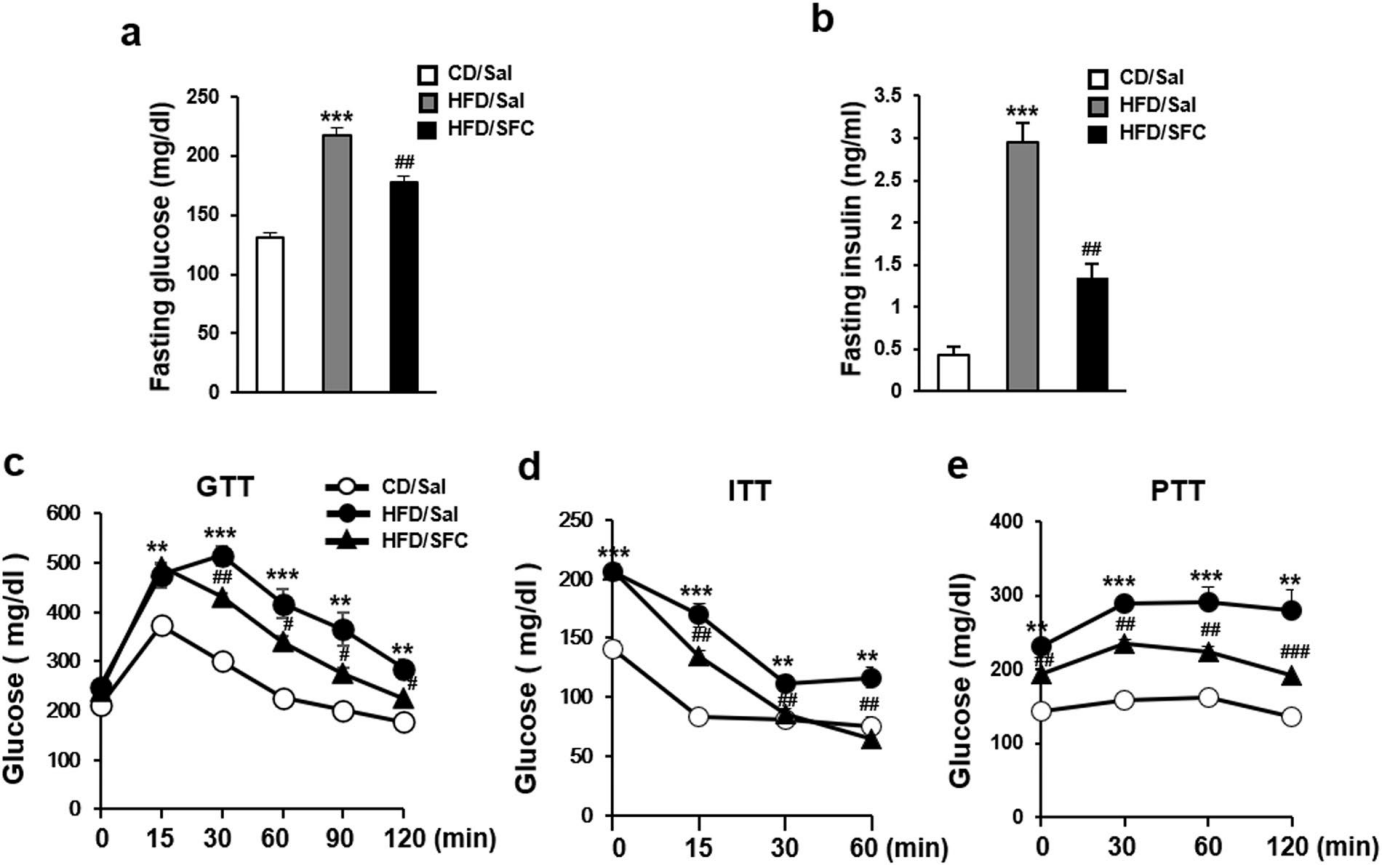
SFC ameliorates HFD-induced hyperglycemia in C57BL/6J mice. Levels of plasma glucose (**a**) and insulin (**b**) after fasting for 6 h were measured using Accu-Check glucometer and insulin ELISA kit, respectively. (**c**) Intraperitoneal glucose tolerance test (GTT) was carried out by measuring glucose levels at 15, 30, 60, 90, and 120 min after peritoneal injection of glucose (1 g/kg) to 6-hr fasted mice. (**d**) Intraperitoneal insulin tolerance test (ITT) was carried out by measuring glucose level at 15, 30, and 60 min after insulin injection (0.7 U/kg) to 6 h fasted mice. (**e**) Intraperitoneal pyruvate tolerance test (PTT) was carried out by measuring glucose level at 30, 60, and 120 min after pyruvate injection (1 g/kg) to 6-hr fasted mice. **p < 0.01; ***p < 0.001 vs. CD mice. ^#^*p* < 0.05; ^##^*p* < 0.01; ^###^*p* < 0.001 vs. HFD mice.

HFD for 15 weeks significantly reduced the levels of insulin-stimulated phospho-AKT in liver tissues (Fig. 4d), suggesting that HFD would induce hepatic insulin resistance. Therefore, the levels of fasting glucose and insulin were initially investigated in HFD-fed mouse plasma. Figure 5a shows that HFD increased the plasma glucose level to 217 mg/dL in 6 h fasting and SFC treatment reduced the level to 178 mg/dL. In addition, HFD increased fasting insulin level by 582% and SFC reduced the increased insulin level in HFD-fed mice by 54.4% (Fig. 5b). These data suggest that HFD for 15 weeks could induce insulin resistance and mild hyperglycemia in C57BL/6J mice, and SFC treatment significantly improved the HFD-induced insulin resistance and hyperglycemia. When glucose tolerance test (GTT) was carried out, HFD group showed higher glucose levels at all time points than CD group, suggesting that HFD induced glucose intolerance. SFC treatment significantly reduced the increased glucose levels in HFD group at most time points except 15 min, suggesting that SFC could improve HFD-induced glucose intolerance (Fig. 5c). In addition, HFD increased glucose levels at all time points compared with CD group in experiments of insulin tolerance test (ITT) and pyruvate tolerance test (PTT). SFC treatment significantly reduced the glucose level at most time points in both ITT and PTT experiments. These data suggest that SFC improves insulin-stimulated glucose clearance in blood and prevents glucose production from pyruvate in liver (Fig. 5d,e). All these data demonstrated that HFD feeding for 15 weeks in C67BL/6J mice induced hyperglycemia, possibly through HFD-induced hepatic insulin resistance and that SFC treatment ameliorated the HFD-induced hyperglycemia, through improvement of the hepatic insulin resistance.

## Discussion

These studies were carried out to determine whether SFC as a fatty acid uptake inhibitor has a protective effect on HFD-induced steatosis and steatohepatitis in C57BL/6J mice. To determine whether liver tissues are sensitive to the inhibitory effect of SFC on fatty acid uptake, incorporation of BODIPY-palmitate as a fluorescence-tagged fatty acid into liver and SFC’s inhibitory effect on the incorporation were initially investigated in C57BL/6J mice. SFC dominantly reduced BODIPY-palmitate incorporation into liver tissues among various tissues. In addition, SFC reduced fasting-induced steatosis induced by excessive mobilization of circulating fatty acids into liver. These data suggest that liver is one of the most sensitive tissues to SFC’s inhibitory effect on fatty acid uptake. Due to SFC having inhibitory activity of fatty acid uptake into hepatocytes, SFC prevented palmitate-induced fat accumulation and lipotoxicity in HepG2 hepatocyte cells. SFC treatment in HFD-fed C57BL/6J mice reduced liver size and triacylglycerol amount in the liver tissues. In company with SFC’s preventing effect on fat accumulation in HFD-fed mouse liver, SFC restored HFD-induced hepatic inflammation and damage. SFC treatment also reduced hepatic insulin resistance, and thus, ameliorated hyperglycemia HFD-fed mice.

SFC is thought to be an intracellular metabolic derivative synthesized from oxaloacetate and sodium fluoroacetate (SFA) by using TCA cycle enzyme citrate synthase, and acts as an allosteric inhibitor of TCA cycle enzyme aconitase^31,32^. Thus, SFC is thought to be a toxic compound to induce energy loss in cells. In fact, high concentration of SFC showed inhibitory effect on aconitase, and thus was cytotoxic to cells^34,35^. However, the concentration of SFC lower than 0.2 mM used in these experiments was suboptimal concentration in showing its inhibitory effect on aconitase, and thus not toxic^33^. In addition, treatment with trans-aconitic acid as another inhibitor of aconitase or knockdown of aconitase was not protective against fatty acid-induced lipotoxicity in beta cells^33^. SFC’s protective effect was highly specific for fatty acid-induced toxicity and another fatty acid uptake inhibitor sulfo-N-succinimidyl oleate had similar protective effect against palmitate-induced cell death^33,36^. In accordance with these reports, our data showed that SFC prevented BODIPY-palmitate uptake and palmitate-induced lipotoxicity in hepatocytes. It is concluded that SFC’s protective effect on fatty acid-induced lipotoxicity in hepatocytes was also due to its inhibitory effect on the cellular uptake of fatty acids.

Liver, muscle, heart, and adipose tissues are the organs having high capacity of taking fatty acid from circulation^37,38^. As expected, liver and adipose tissues showed high capacity of fatty acid intake, but muscle and heart showed low capacity of intake of fatty acid in our experiments. It was supposed that fatty acid uptake in muscle and heart seemed to be lower, since we investigated fatty acid uptake by measuring BODIPY fluorescent intensity per mg protein, and muscle and heart were tissues having a high content of protein. When fatty acid uptake was investigated in culture cells, the inhibitory effect of SFC on fatty acid uptake was observed only in HepG2 hepatocytes, but not in C2C12 muscle and 3T3L1 adipose cells. In addition, the inhibitory effect of SFC on fatty acid uptake was significant in liver tissue among various tissues. The reason why SFC has high inhibitory effect on fatty acid uptake in liver tissues was not determined. Different expression of fatty acid transporters and fatty acid binding proteins in tissues may explain the specificity of SFC’s inhibitory effect on fatty acid uptake into hepatocytes^39,40^. In fact, SFC treatment showed selective reduction in expression levels of molecules related to fatty acid transport. SFC slightly reduced the protein levels of fatty acid translocase CD36 and RNA levels of fatty acid transport-related genes such as CD36, FATP5, and FABP2 in HepG2 cells (Supplementary Fig. 2a,b of SI).

Since SFC inhibits fatty acid uptake into hepatocytes, it is supposed that SFC treatment prevents palmitate-induced fat accumulation and lipotoxcity in hepatocytes. Treatment with non-toxic low concentration of palmitate (0.1 mM) for 24 h induced fat accumulation in HepG2 hepatocytes without the induction of cell death. Nile red staining and triacylglycerol assay demonstrated that SFC could prevent palmitate-induced lipid accumulation in hepatocytes. SFC also reduced fat accumulation induced by oleic acid as an unsaturated fatty acid in HepG2 cells (Supplementary Fig. 3 of SI), suggesting that SFC’s inhibitory effect on fatty acid uptake does not differentiate the saturation of fatty acids. On the other hand, treatment with high concentration (0.3 mM) of saturated fatty acid palmitate for 15 h induced cell death in HepG2 cells. SFC significantly prevented the palmitate-induced viability reduction, DNA fragmentation and caspase 3 cleavage in these cells. Fatty acid-induced lipotoxicity in hepatocyte was reported to be mediated by excessive activation of stress phospho-JNK signals induced through oxidative and/or endoplasmic reticulum stress or by activation of inflammatory phospho-NFκB signals^41–43^. SFC clearly reduced levels of palmitate-induced phospho-JNK and phospho-p65 in HepG2 cells. In conjunction with the reduction of stress/inflammatory signals, SFC treatment reduced the palmitate-induced expression of inflammatory genes such as MCP1, TNFα, and IL-1β. These data suggest that the activation of stress/inflammatory signals by palmitate treatment is attributed to excessive influx of fatty acids and metabolic derangement of the fatty acids in hepatocytes. Mitochondrial dysfunction by metabolic derangement of fatty acids and subsequent activation of stress/inflammatory signals was reported to play a key role in fatty acid-induced lipotoxicity^44–46^.

While HFD increased body weight in C57BL/6J mice, treatment with SFC prevented the HFD-induced body weight increase without change of food intake. Accompanying the reduction of body weight, SFC treatment significantly decreased weight of liver (Fig. 3b). However, SFC treatment did not affect the weight of white adipose fats, and even increased the weight of epididymal fat in HFD-fed mice. Less mobilization of fatty acids into liver from adipose tissues due to the SFC’s inhibitory effect on fatty acid uptake into hepatocyte but negligible effect of SFC on fatty acid uptake into adipose tissue might explain the decreased weight of liver but increased weight of white adipose tissues in SFC-treated mice (Figs. 1d and 3b). On the other hand, SFC treatment significantly restored HFD-induced stress/inflammatory signal activation and inflammatory gene expression in HFD-fed C57BL/6J mouse liver. SFC also prevented HFD-induced increase of plasma AST and ALT. These data suggest that SFC treatment could improve both HFD-induced steatosis and steatohepatitis, mainly though less mobilization of fatty acids to liver due to its inhibitory activity of fatty acid uptake into hepatocyte. These results also support the hypothesis that HFD-induced NASH could be developed due to the toxic lipid derivatives formed through augmented intake of fatty acids into liver^3^. However, it cannot be excluded that SFC ameliorates HFD-induced NAFLD through inhibition of lipid synthetic metabolism or activation of lipid degradation metabolism.

In company with the improvement in hepatic stress/inflammatory signals, SFC improved HFD-induced downregulation of hepatic insulin signals. Since insulin signal cascades including phospho-AKT and phospho-GSK3b were reported to be blunted by several serine kinases, such as JNK and IKK^47–49^, SFC’s improving effect on stress/inflammatory signals would improve the blunted signals in insulin downstream signal cascades. As expected, phospho-AKT and phospho-GSK3b reduced in HFD-fed mouse liver were restored by SFC treatment. In company with the improvement in insulin signaling pathway in palmitate-treated hepatocytes, SFC treatment improved HFD-induced hepatic insulin resistance. SFC reduced the fasting level of serum insulin and improved intolerance of glucose, insulin, and pyruvate (Fig. 5b,d,e). Since NAFLD is considered a manifestation of metabolic syndrome^50^, it is reasonable that SFC treatment would improve HFD-induced NAFLD through SFC’s improving effect on hepatic insulin resistance. In company with improvement of hepatic insulin resistance, SFC treatment ameliorated hyperglycemia in HFD-fed mice.

In conclusion, SFC showed inhibitory effect on fatty acid uptake into hepatocytes, and thus preventive effect on fatty acid-induced lipotoxicity in the hepatic cells. While short-term treatment with SFC prevented fasting-induced hepatic steatosis in C57BL/6J mice, long-term treatment with SFC reduced HFD-induced hepatic steatosis, inflammation, and injury in the same mice. In addition, SFC improved HDF-induced hepatic insulin resistance and hyperglycemia. These data suggest that the reduction of fatty acid mobilization into liver from circulation through treatment with a fatty acid uptake inhibitor would be a promising strategy to prevent HFD-induced NAFLD.

## Methods

### Ethics statement

All experiments were performed in accordance with the Ajou University Safety and Ethics guidelines. In particular, animal experiments were carried out according to the animal experiment procedure approved by the Animal Ethics Committee of Ajou University.

### Materials

Most chemicals used in this study were purchased from Sigma–Aldrich (St. Louis, MO, USA) including the followings: glucose, palmitate, 3-[4,5-dimethylthiazol-2-yl]-2,5-diphenyltetrazoilium bromide (MTT), DL-fluorocitric acid barium salt, Oil Red O and Eosin Y. Nile Red and Hematoxylin. BODIPY™-palmitate (4,4-Difluoro-5,7-Dimethyl-4-Bora-3a,4a-Diaza-*s*-Indacene-3-Hexadecanoic Acid) was obtained from Thermo Fisher Scientific (Waltham, MA, USA). Anti-caspase 3, anti-phospho-AKT, anti-total AKT, anti-phospho-GSK3b, anti-total GSK3b, anti-phospho-JNK, anti-total JNK, anti-phospho-P65, and anti-total P65 antibodies were obtained from Cell Signaling Technology (Beverly, MA, USA). Anti-actin and anti-tubulin antibodies were purchased from Bethyl Laboratory (Montgomery, TX, USA) and Santa Cruz Biotechnology (Dallas, TX, USA), respectively. The catalog numbers of all reagents and antibodies were listed in Supplementary Table 2 of SI.

### Cells and culture

Human liver cancer cell HepG2 was obtained from Korean Cell Line Bank, and maintained in DMEM medium supplemented with 10% FBS (Capricon Scientific, Ebsdorfergrund, Germany), 100 U/ml penicillin (Duchefa Biochemie, Haarlem, Netherlands), and 100 μg/ml streptomycin (Duchefa Biochemie) at 37 °C in a humidified atmosphere containing 5% CO_2_.

### BODIPY-palmitate uptake test in mouse tissues

A total of twelve 8-week-old male mice were assigned to three groups: Saline (n = 4); BODIPY-palmitate/saline (n = 4); and BODIPY-palmitate/SFC (n = 4). To assess the inhibitory effect of SFC on fatty acid uptake in different tissues, mice were fasted 12 h and then intraperitoneally injected with Saline or SFC (20 mg/kg). One hour after SFC administration, BODIPY-palmitate (50 μg/mouse) were intravenously administered into the BODIPY groups. Animals were sacrificed, and various tissues were then isolated at 2 h after BODIPY-palmitate administration. The extracts were obtained by dissolving the tissues in RIPA buffer (150 mmol/L NaCl, 1% NP-40, 0.5% deoxycholate, 0.1% sodium dodecyl sulfate, 50 mmol/L Tris.HCl at pH 7.5) and subsequent centrifugation (10,000xg, 10 min). The relative uptake of BODIPY-palmitate was determined by measuring the fluorescence intensity per mg of protein by fluorescence spectrophotometry (PerkinElmer Victor X3, Waltham, MA, USA) at 430 nm and 510 nm as exciting and emitting wavelength, respectively.

### Palmitate uptake assay in HepG2 cells

Palmitate uptake was determined by measuring relative fluorescence intensity in cells after treatment with BODIPY-palmitate. Briefly, cells grown in 96-well plate were washed with PBS buffer (137 mM NaCl, 2.7 mM KCl, 10 mM Na_2_HPO_4_, 1.8 mM KH_2_PO_4_) and then incubated in 100 μl of 2.0 μM BODIPY-palmitate at 37 °C for 3 h. After washing with PBS three times, 100 μl of new PBS was overlaid onto the cells. The fluorescence in each well was measured using fluorescence spectrophotometry (PerkinElmer Victor X3,) at 430 nm and 510 nm as exciting and emitting wavelength, respectively. The uptake of fatty acid was determined by counting the relative intensity of fluorescence.

### Immunoblotting

RIPA buffer including protease inhibitor cocktail (Roche Applied Science) was used to extract cellular proteins. Equivalent amounts of protein (30 μg) in sodium dodecyl sulfate (SDS) sample buffer (50 mmol/L Tris-HCl at pH 6.8, 2% SDS, 100 mmol/L DL-dithiothreitol, 10% glycerol) were separated by SDS-polyacrylamide gel electrophoresis (PAGE), and then transferred to polyvinylidene difluoride (PVDF) membrane (Millipore, Bedford, MA). After blocking the PVDF membranes with 5% skimmed milk for 30 min, target antigens were reacted with primary antibodies. After binding with secondary antibodies (horseradish peroxidase-conjugated anti-mouse IgG or anti-rabbit IgG antibodies), immunoreactive bands were detected with enhanced chemiluminescence system (Pierce, Rockford, IL, USA). Band intensities were determined with densitometric analysis using one-dimensional Quantity One^®^ 1D image analysis system (Bio-Rad, Hercules, CA).

### Reverse transcriptase-polymerase chain reaction

Expression levels of mRNAs were determined using a semi-quantitative reverse transcriptase-polymerase chain reaction (RT-PCR) kit supplied by Takara RNA PCR kit Ver 3.0 (Takara, Shiga, Japan) and RT-PCR machine (TP850, Takara). Briefly, total RNAs were extracted with TRIzol (Invitrogen, Carlsbad, CA). RNA quantity and purity were determined by NanoDrop 1000 spectrophotometry (Thermo Fisher Scientific, Waltham, MA). cDNAs were synthesized with AMV reverse transcriptase and random 9-mers and used as template for PCR amplification. The primer sets for amplification were listed in Supplementary Table 3 of SI. DNAs were amplified under the following conditions: denaturation at 95 °C for 5 min followed by 26 cycles of denaturation at 95 °C for 30 sec, annealing at 60 °C for 30 sec, and extension at 72 °C for 1 min. Relative quantity of amplified DNAs was analyzed using software supplied from TP850.

### Animal studies

A total of twenty-six 6-week-old male C57BL/6 mice were obtained from Japan SLC Inc (Shizuoka Ken, Japan). Mice were housed in a temperature-controlled room at 22 ± 2 °C with a light/dark cycle of 12 h and fed *ad libitum*. Following two weeks of adaptation, mice were randomly assigned into the following three groups: 1) saline-injected control diet group (CD/Sal) (n = 6); 2) saline-injected high fat diet group (HFD/Sal) (n = 10); and 3) SFC-injected HF diet group (HFD/SFC) (n = 10). For the control diet group, mice were fed normal chow diet containing 10% fat (D12450B; Research Diets Inc., New Brunswick, NJ) and water. For the HFD group, mice were fed chow diet with 60% fat (D12492; Research Diets Inc.) and water. Treated groups were injected intra-peritoneally every other day with saline or 10 mg/kg of SFC for 15 weeks. Weight of mice and consumed diet were measured every week. Blood was collected from tail. Glucose levels were measured using Accu-check (Korea Roche Diagnostics, Seoul, Korea). All animal care and treatments were conducted according to the Ajou Institutional Animal Care guidelines and were approved by the Ajou Institutional Animal Care Committee (Permission Number: 2018-0046).

### Statistical analysis

All experiments were repeated at least three times. Data are expressed as mean ± SE. All data were analyzed by GraphPad Prism 6.0 (GraphPad software, San Diego, CA). One-way analysis of variance (ANOVA) with Bonferroni post hoc test was used to obtain statistical significance. Statistical significance was accepted at p < 0.05.

The Supplementary Methods of Supplementary Information (SI) include methods for the preparation of palmitate, preparation of sodium fluorocitrate, viability assay, DNA fragmentation assay, insulin measurement, glucose tolerance test, insulin tolerance test. pyruvate tolerance test, measurement of alanine aminotransferase (ALT) and aspartate aminotransferase (AST), measurement of triacylglyceride, Oil Red O staining, F4/80 staining, and Nile Red staining.

## Supplementary information

Sodium fluorocitrate having inhibitory effect on fatty acid uptake ameliorates high fat diet-induced non-alcoholic fatty liver disease in C57BL/6J mice

## Data Availability

The materials and datasets generated during the current study are available from the corresponding author on reasonable request.
