# Supplementary material for: Sodium fluorocitrate having inhibitory effect on fatty acid uptake ameliorates high fat diet-induced non-alcoholic fatty liver disease in C57BL/6J mice
Source: Sci Rep. 2019 Nov 28;9:17839. doi: 10.1038/s41598-019-54476-5 (PMC6882787; doi:10.1038/s41598-019-54476-5)
Supplement: Supplementary file 1 — Sodium fluorocitrate having inhibitory effect on fatty acid uptake ameliorates high fat diet-induced non-alcoholic fatty liver disease in C57BL/6J mice [file 41598_2019_54476_MOESM1_ESM.pdf]

Supplementary Information

Sodium fluorocitrate having inhibitory effect on fatty acid uptake ameliorates high fat diet-induced non-alcoholic fatty liver disease in C57BL/6J mice

Seung A Hong, Ik-Rak Jung, Sung-E Choi, Yoonjung Hwang, Soo-Jin Lee, Youngho Son, Yu Jung Heo, Rihua Cui, Seung Jin Han, Hae Jin Kim, Kwan Woo Lee, Yup Kang

Supplementary Methods

Preparation of palmitate. Palmitate/bovine serum albumin (BSA) conjugates were prepared by soaping palmitate with sodium hydroxide and mixing with BSA. Palmitate (20 mmol/L in 0.01 mol/L NaOH) was incubated at 70 °C for 30 min. These fatty acid soaps were then complexed with 5% BSA in phosphate-buffered saline (PBS) in a 1:3 volume ratio. Complexed palmitate/BSA conjugates consisted of palmitate (5 mmol/L) and BSA (3.75%) were diluted in culture medium with appropriate concentration, and then administered to cultured cells.

Preparation of sodium fluorocitrate. Sodium fluorocitrate was prepared from barium fluorocitrate (Sigma-Aldrich). One hundred mg of fluorocitric acid barium in 1.2 ml of deionized water was dissolved by the addition of 60.58 μL of 37% HCl. Anhydrous sodium sulfate (51.12 mg) and anhydrous sodium carbonate (38.16 mg) were sequentially added to the fluorocitric acid solution. Insoluble barium sulfate was removed by centrifugation (10,000xg, 10 min), and upper sodium fluorocitrate solution was then collected in new tube. The sodium fluorocitrate solution was prepared at a final concentration of 0.1 M at pH = 7 ± 1.

Viability assay. Briefly, 100 μL of MTT (0.5 mg/mL) solution was added to cells in 96-well plate, and then incubated at 37 °C for 1 h. Upper medium was discarded, and 100 μL of acidic isopropanol (0.04 mol/L HCl) were then added to each well. After incubating at room

temperature for 30 min, absorbency was measured at 570 nm by a microplate reader (BIO-RAD, Hercules, CA, USA).

DNA fragmentation assay. Cell death was determined by measuring fragmented DNAs using Cell Death Detection enzyme-linked immunosorbant assay (ELISA^{plus}) kit (Roche Applied Science, Mannheim, Germany), according to the manufacturer's instructions. Briefly, cells were lysed by adding lysis buffer supplied with the kit. After centrifugation (200xg, 10 min), the supernatant was pipetted onto an anti-streptavidin-coated microplate. Anti-DNA monoclonal antibody conjugated with peroxidase (anti-DNA-POD) and anti-histone-biotin was serially added. After incubation for 90 min, wells were rinsed with incubation buffer three times. The color was developed by reaction with 2,20-azino-di-[3-ethylbenzthiazoline sulphonate] (ABTS) substrate solution for 10–20 min. The amount of peroxidase retained in the nucleosome complex was determined by measuring the absorbance at 405 nm by a microplate reader (BIO-RAD, Hercules, CA, USA).

Insulin measurement. Plasma insulin was measured using Shibayagi Mouse Insulin ELISA kit (Cunma, Japan). Briefly, blood obtained from mouse tail was immediately centrifuged at 3,000xg for 10 min at 4 °C. Upper plasma was collected and stored at -80 °C. One hundred microliter of biotinylated-anti-insulin antibody solution placed in each well of 96-well plate coated with anti-insulin was mixed with 10 μL of plasma sample. After 2 h incubation at room temperature, the complex solution between insulin and biotinylated-anti-insulin antibody was removed. The biotinylated-anti-insulin antibody bound to insulin coated on plate was incubated with 100 μL of HRP-streptavidin solution supplied from kit for 30 min at room temperature. After removing unbound HRP-streptavidin, the HRP-streptavidin bound

on plate was reacted with 3, 3', 5, 5' – Tetramethylbenzidine (TMB) in 100 µL chromogen solution. After stop reaction with 100 µL of 1 M sulfuric acid, the absorbency at 450 nm was measured using a microplate reader (BIO-RAD). The amount of insulin in plasma was calculated using an insulin standard curve.

Glucose, insulin, and pyruvate tolerance test. Mice were first subjected to 6 h fasting and injected intraperitoneally with glucose (1 g/kg), insulin (0.7 U/kg), or pyruvate (1 g/kg) for glucose tolerance test (GTT), insulin tolerance test (ITT), or pyruvate tolerance test (PTT), respectively. Blood was collected from tail at 0, 15, 30, 60, 90, and 120 min after glucose infusion; at 0, 15, 30, and 60 min after insulin injection; and at 0, 30, 60, and 120 min after pyruvate injection. Glucose levels (mg/dL) in blood were then measured by using Accu-chek (Korea Roche Diagnostics, Seoul, Korea).

Measurement of alanine aminotransferase (ALT) and aspartate aminotransferase (AST) in plasma. Blood obtained from mouse heart was immediately centrifuged at 1,500xg for 15 min at 4 °C. Upper plasma was collected and stored at -80 °C. Plasma levels of ALT and AST were measured using ALAT/GPT and ASAT/GOT kit (Roche Diagnostic International, Mannheim, Germany), according to the manufacturer's instructions. Briefly, pyruvate and oxaloacetate were produced from alanine and aspartate by using ALT and AST, respectively. NADH reductions during conversion from pyruvate and oxaloacetate to lactate and malate by using lactate dehydrogenase and malic enzyme, respectively, were measured by autochemical analyzer (Hitachi 7600, Tokyo, Japan). Reduction of NADH is proportional to ALT and AST activity.

Measurement of triacylglyceride. Tissue or cell triglyceride was extracted using the Folch extraction method (*J Biol Chem* **226,** 497–509. 1957), and the amount was then measured with commercially available kit (Biovision, Triglyceride Quantification Colorimetric/Fluorometric Kit K622, Milpitas, CA), according to the manufacturer's instructions. Briefly, cells or tissues in 5% NP40 solution were homogenized and then boiled for 5 min. Five microliter of extract obtained by centrifugation (10,000xg for 2 min) was diluted with 130 μL of TG assay buffer supplied from kit to make TG solution. Fifty microliter of TG solution was reacted with 2 μL of lipase (kits) at room temperature for 20 min, and the digested TG solution was then mixed with 50 μL of TG reaction mix supplied from kit. After incubation at room temperature for 60 min, the absorbance was measured at 570 nm by a microplate reader (BIO-RAD, Hercules, CA, USA). The amount of TG was calculated using standard curve.

Oil Red O staining. Existence of lipid droplet in liver tissues was determined by Oil Red O staining. Liver tissue frozen in OCT compound was cut into 5 μm thickness, mounted onto slides, fixed with 10% formalin for 30 minutes, soaked in 60% isopropanol solution for 5 minutes, and stained in Oil Red O solution (0.5% in 60% isopropanol solution) for 10 minutes. After rinsing the sections three times in water, nuclei were stained by soaking the tissues in Hematoxylin solution (Abcam, Cambridge, UK) for 1 min. Red oil drops were observed under by Aperio Scan Scope CS (Leica, Wetzlar, Germany).

Nile Red staining. Accumulation of neutral lipid in HepG2 cells by palmitate treatment was determined by Nile Red staining kit (Abcam 228553, Cambridge, UK). HepG2 cells were treated with 0.1 mM palmitate for 24 hrs. Cells were incubated in Nile Red solution supplied

by kit for 10 min after aspiration of media and washing three times with DMEM media. After removing the Nile Red solution, the stained lipids in cells were observed by confocal microscopy (Zeiss LSM 710) at 540 nm as excitation and 630 nm as emission wavelength.

F4/80 staining. Macrophages in liver tissues were detected by F4/80 staining. Frozen liver tissues sections were blocked with 5% normal bovine serum. EGF-like module-containing mucin-like hormone receptor-like 1 antigen (F4/80) were reacted with anti-mouse F4/80 primary antibody and horse radish peroxidase (HRP)-conjugated rabbit anti-rat IgG secondary antibody. Brown colors were detected using 3,3'-diaminobenzidine (DAB) as HRP substrate supplied from Kit (Vector DAB peroxidase substrate kit SK-4100, Burlingame, CA, USA), according to the manufacturer's instructions.. Nuclei were stained by incubation of the sections in Hematoxylin solution (Abcam, Cambridge, UK) for 1 min. Brown colored macrophages with small violet nuclei were detected and counted in tissue images (Aperio ScanScope CS).

Supplementary Tables.

Table 1. NAFLD Activity Score (NAS)

	Animal #	Steatosis	Inflammation	Ballooning	Sum	NAS (Average)	S.E.
**CD**	N1	1	0.3	0	1.3	**0.783333**	0.282154
	N2	0	0	0	0		
	N3	0	0	0	0		
	N4	1	0	0	1		
	N5	1	0.7	0	1.7		
	N6	0.7	0	0	0.7		
	Average	0.61666667	0.166666667	0	0.783333		
**HFD**	H1	2.7	2	1	5.7	**5.2**	0.192065
	H2	3	0.3	1.3	4.6		
	H3	3	1	1	5		
	H4	2	1	1	4		
	H5	3	1	1	5		
	H6	3	1.7	1	5.7		
	H7	3	1	1	5		
	H8	3	1.7	1	5.7		
	H9	3	1.3	1	5.3		
	H10	3	2	1	6		
	Average	2.87	1.3	1.03	5.2		
**HFD/SFC**	S1	1	0	0	1	**1.93**	0.331344
	S2	1.3	0.3	0	1.6		
	S3	1.3	0	0	1.3		
	S4	1	0	0	1		
	S5	1	0.7	0.7	2.4		
	S6	2	0.3	1	3.3		
	S7	0.3	0.3	0	0.6		
	S8	1	0	0.7	1.7		
	S9	1.7	1	1	3.7		
	S10	1	1	0.7	2.7		
	Average	1.16	0.36	0.41	1.93		

Table 2. Catalog numbers and company names of reagents and antibodies

Reagents	Catalog Number	Company
Glucose	G8769	Sigma-Aldrich (St.Louis, MO, USA)
Palmitate	P5585	
DL-fluorocitric acid barium salt	F9634	
Oil Red O	O0625	
Eosin Y	230251	
Hematoxylin Solution, Harris Modified	HHS32	
Nile Red staining kit	Ab228553	Abcam (Cambridge, UK)
3-[4,5-dimethylthiazol-2-yl]-2,5-diphenyltetrazoilium bromide (MTT)	M6494	Thermo Fisher Scientific (Waltham, MA, USA)
BODIPY™ FL C16	D3821	
Anti-cleaved caspase3 antibody	9661	Cell Signaling Technology (Beverly, MA, USA)
Anti-phospho-AKT antibody	9271	
Anti-total AKT antibody	9272	
Anti-phospho-GSK3b antibody	9331	
Anti-total GSK3b antibody	9315	
Anti-phospho-JNK antibody	9251	
Anti-total JNK antibody	9252	
Anti-phospho-P65 antibody	3033	
Anti-total P65 antibody	3034	
Anti-actin antibody	A300-491A	Bethyl laboratories (Montgomery,TX, USA)
Anti-tubulin antibody	5286	Santa Cruz Biotechnology (Dallas, TX, USA)
Anti-F4/80 antibody	14-4801-82	Thermo Fisher Scientific (Waltham, MA, USA)
Fetal Bovine Serum	FBS-22A	Capricon Scientific (Ebsdorfergrund, Germany)
100 U/ml penicillin	P0142	Duchefa Biochemie (Haarlem, Netherlands)
100 μg/ml streptomycin	S0148	
Protease inhibitor cocktail	11 836 170 001	Roche Applied Science (Mannheim, Germany)
RNAiso Plus	9109	Takara (Shiga, Japan)
9-mers	3801	Takara (Shiga, Japan)
AMV reverse transcriptase	3001	Beams Biotechnology (Seongnam-si, Gyeonggi-do, Korea)
Rodent Diet With 10 kcal% Fat	D12450B	Research Diets Inc. (New Brunswick, NJ, USA)
Rodent Diet With 60 kcal% Fat	D12492	
Saline	0.9% NS	JW Life Science (Dangjin-si, Chungcheongnam-do, Korea)
Triglyceride Quantification Colorimetric/Fluorometric Kit	K622	Biovision (Milpitas, CA, USA)
Cell Death Detection enzyme-linked immunosorbant assay (ELISAplus) kit	11920685001	Roche Applied Science (Mannheim, Germany)
Mouse Insulin ELISA Kit	638-01489	Shibayagi (Cunma, Japan)
Immobilon-P PVDF Membrane	IPVH00010	Millipore (Bedford, MA, USA)

Table 3. Primer sequences used for quantitative real time-PCR

Gene	GenBank Accession No	Forward (5'-3')<br>Reverse (5'-3')	Amplicon size
SREBF	NM_0011480.4	GCTGTTGGCATCCTGCTATC<br>AGCTGGAAGTGACGGTGGT	174
FASN	NM_007988.3	CCTGGATAGCATTCCGAACCT<br>AGCACATCTCGAAGGCTACACA	122
ACACA	NM_133360.2	ACATCCCCACGCTAAACAGA<br>GTGCAACTAGGAACGTAAGT	420
SCD1	NM_009127.4	CACACGCCGACCCTCACAAT<br>TTTGACAGCCGGGTGTTTGC	86
MCP-1	NM_011333.3	CAGCCAGATGCAGTTAACGC<br>GCCTACTCATTGGGATCATCTTG	73
TNF-α	NM_013693.2	AGCCCCCAGTCTGTATCCTT<br>GGTCACTGTCCCAGCATCTT	113
IL-1β	NM_008361.3	TCTCGCAGCAGCACATCAACA<br>CCTGGAAGGTCCACGGGAAA	105
IL-6	NM_001314054.1	CCATCCAGTTGCCTTCTTGGG<br>GCCGTGGTTGTCACCAGCAT	45
CD68	NM_001291058.1	AGGGACACTTCGGGCCATGT<br>GGGTGATGCAGAAGGCGATG	120
F4/80	NM_001355722.1	CCCGTGTTGTTGGTGGCACT<br>GCTTTGGCTGGATGTGCTGG	90
L3T4	NM_013488.3	AGGAAGTGAACCTGGTGGTG<br>CTCCTGCTTCAGGGTCAGTC	107
NE	NM_015779.2	AGGTGGTCATTATGGCTTCG<br>ATTGTACCCAGATGCCTTCG	81
RPL32	NM_172086.2	AAGCGAAACTGGCGGAAACC<br>CCCATAACCGATGTTGGGCA	90

Supplementary Figures

Supplementary Figure 1

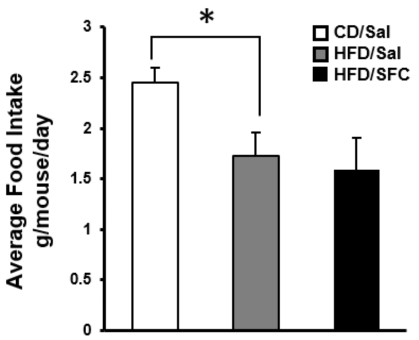

Amount of food intake per mouse every day. Mice were fed with control chow diet (CD) or high fat diet for 15 weeks (HFD).SFC (10 mg/kg) or saline (Sal) was injected into mice every other day for 15 weeks. Consumed food was measured every week and amount of food intake per day was calculated.

Supplementary Figure 2

a.

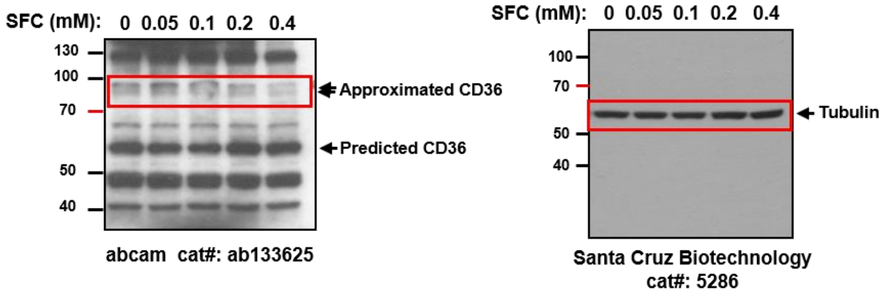

b.

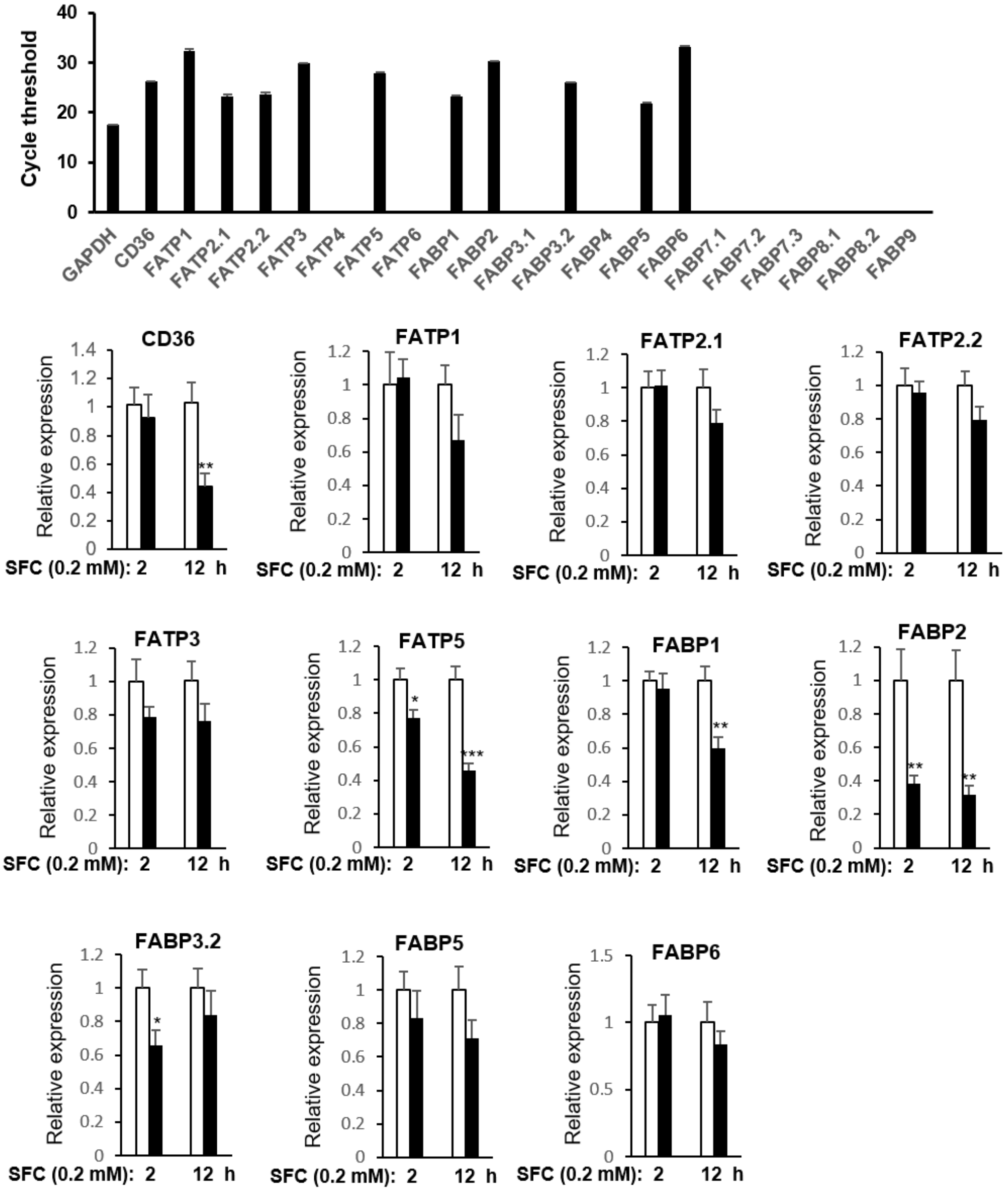

c. Primer sets for genes involved in fatty acid transport

Gene	GenBank Accession No	Forward (5'-3')<br>Reverse (5'-3')	Amplicon size
CD36	NM_ 001371075.1	CCACAGCCAGATTGAGAACTGTGA<br>TTTCCTGCAGCCCAATGGTG	89
FATP1	NM_ 198580.2	GGGTCAGATCAACCAACAGG<br>ACATGTAGCCCAGCTCATCC	155
FATP2.1	NM_003645.3	GGAACCACAGGTCTTCCAAA<br>TTAGTCCGCAAGGCAAGAGT	194
FATP2.2	NM_001159629.1	CTGGTGTCGCCAGAACTACA<br>TTAGTCCGCAAGGCAAGAGT	248
FATP3	NM_024330.2	CCAGTACATTGGGGAGCTGT<br>CCGCTGTCCTGTGTAGTTGA	208
FATP5	NM_012254.2	AGCTCCTGCGGTACTTGTGT<br>CTGTGGAGCCGTAGACTTCC	156
FABP1	NM_001443.2	GCAGAGCCAGGAAAACTTTG<br>TCTCCCCTGTCATTGTCTCC	206
FABP2	NM_ 000134.3	TTGGAAGGTAGACCGGAGTG<br>AGGTCCCCCTGAGTTCAGTT	230
FABP3.2	NM_004102.4	GGTGTGGGTTTTGCTACCAG<br>GGTGAACAAGTTTCCCTCCA	211
FABP5	NM_001444.2	ATGGCCAAGCCAGATTGTAT<br>TGAACCAATGCACCATCTGT	176
FABP6	NM_ 001040442.1	GGCAAGTTCGAGATGGAGAG<br>ACAGTGGCCTTGAACGTCTT	239

Down-regulation of genes involved in fatty acid transport by SFC treatment in HepG2 cells.

HepG2 cell were treated with 0.2 mM SFC for 12 h. (a) Immunoblotting for CD36 and tubulin was carried out using anti-CD36 antibody (Abcam, Cambridge, UK) and anti-tubulin antibody (Santa Cruz, Dallas, TX) a primary antibody, respectively. (b) HepG2 cells were treated with 0.2 mM SFC for 2 h or 12 h. RNA was extracted with Trizol and cDNAs were then synthesized with AMV reverse transcriptase. DNAs were amplified using Takara PCR kit Ver 3.0 with primer sets under the following condition: denaturation at 95 °C for 5 min, followed by 26 cycles of denaturation at 95 °C for 30 sec, annealing at 60 °C for 30 sec, and extension at 72 °C for 1 min. Cycle threshold (Ct) value of all genes and relative quantity of amplified DNAs were analyzed using software supplied by TP850. (c) Primer sets for genes involved in fatty acid transport.

Supplementary Figure 3

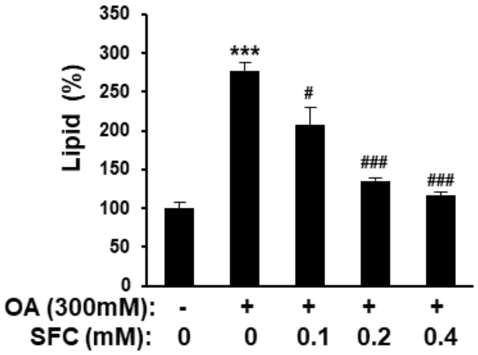

Inhibitory effect of SFC on oleic acid-induced lipid accumulation in HepG2 cells. Lipid accumulation in HepG2 hepatocytes was induced by treatment with 0.3 mM oleic acid as unsaturated fatty acid for 16 hrs. Neutral lipid in cells was stained with BODIPY 493/503 (Thermo Fisher Scientific) for 3 hrs. The fluorescence intensity was measured using fluorescence spectrophotometry (PerkinElmer) at 480 as excitation and 510 nm as emission, and the relative amount of lipid was calculated.

Supplementary Figure 4

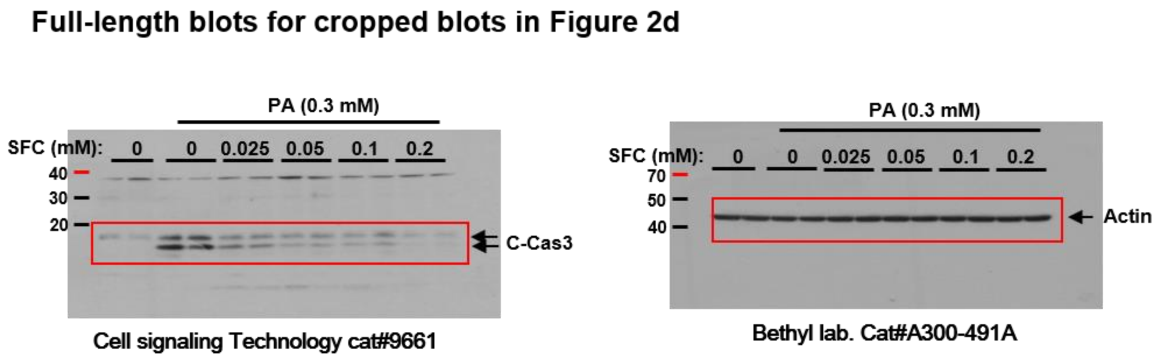

Whole blot images of western blotting analysis for cleaved caspase 3 (C-Cas3) and actin in Figure 2d. Cropped blots were designated by delineation with red dividing lines. The molecular weights are indicated at left of ladders. Supplier and catalogue number of antibodies are also written below images.

Supplementary Figure 5

Full-length blots for cropped blots in Figure 2e

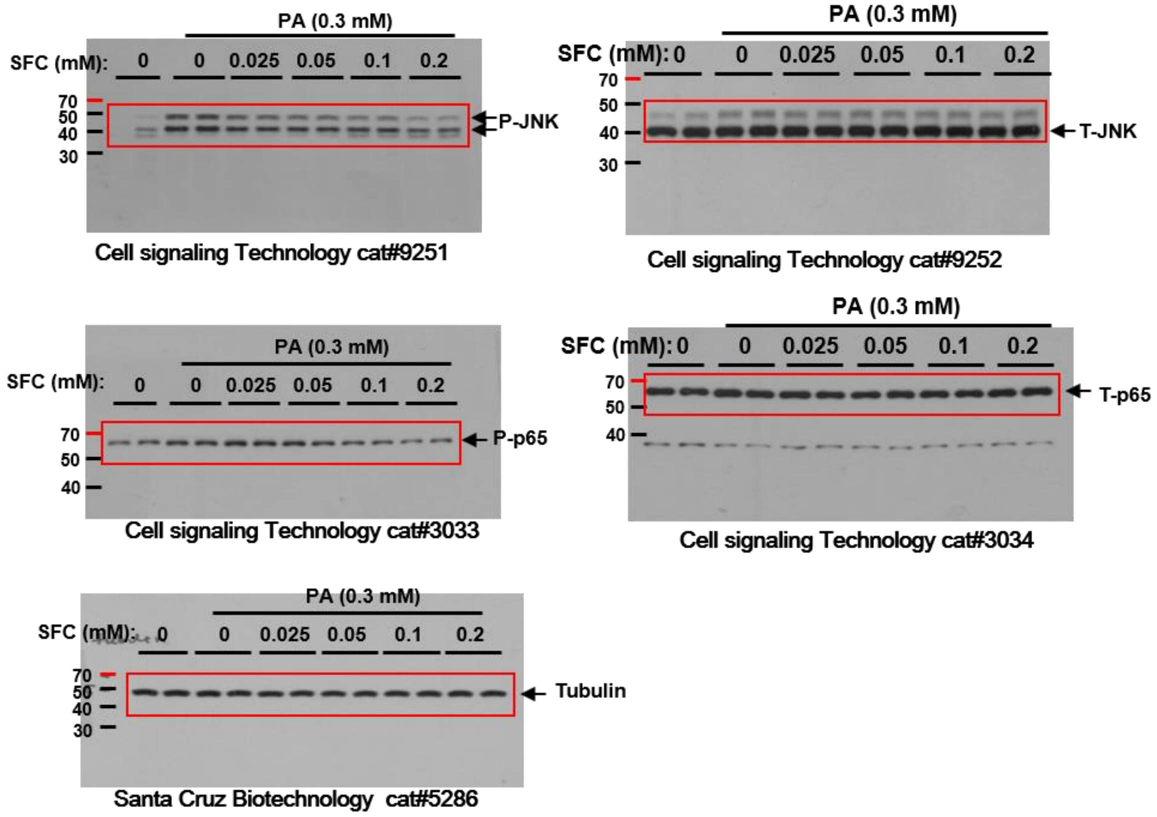

Whole blot images of western blotting analysis for phospho-C-JUN-N-terminal kinase (P-JNK), total-JNK, phospho-p65 subunit of NFκB (P-p65), total-p65 (T-p65), and tubulin in Figure 2e. Cropped blots were designated by delineation with red dividing lines. The molecular weights are indicated at left of ladders. Supplier and catalogue number of antibodies are also written below images.

Supplementary Figure 6

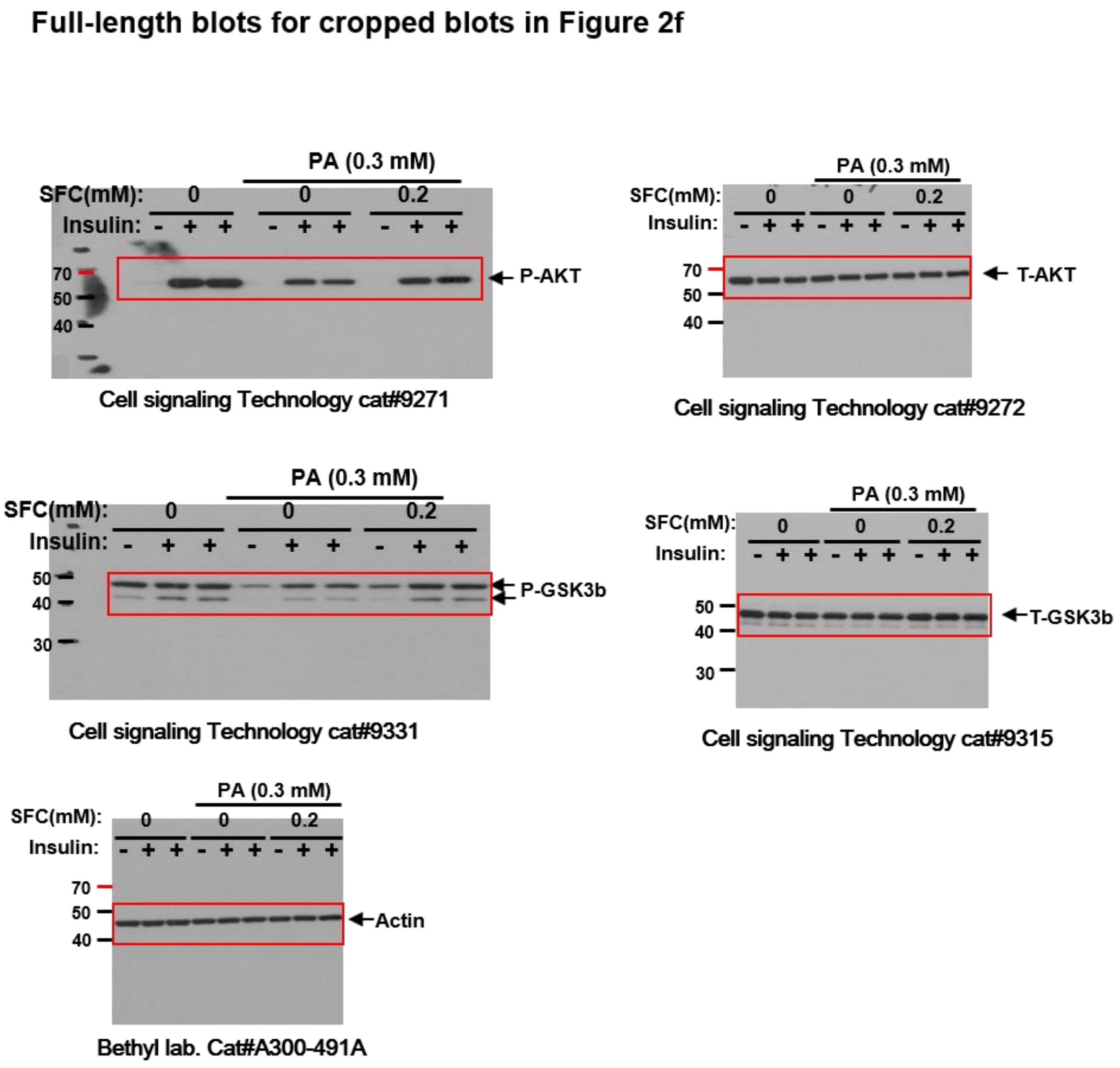

Whole blot images of western blotting analysis for cleaved phospho-AKT (P-AKT), total AKT, phospho-GSK3b (P-GSK3b), total-GSK3b (T-GSK3b) and actin in Figure 2f. Cropped blots were designated by delineation with dividing lines. The molecular weights are indicated at left of ladders. Supplier and catalogue number of antibodies are also written below images.

Supplementary Figure 7

Full-length blots for cropped blots in Figure 4c

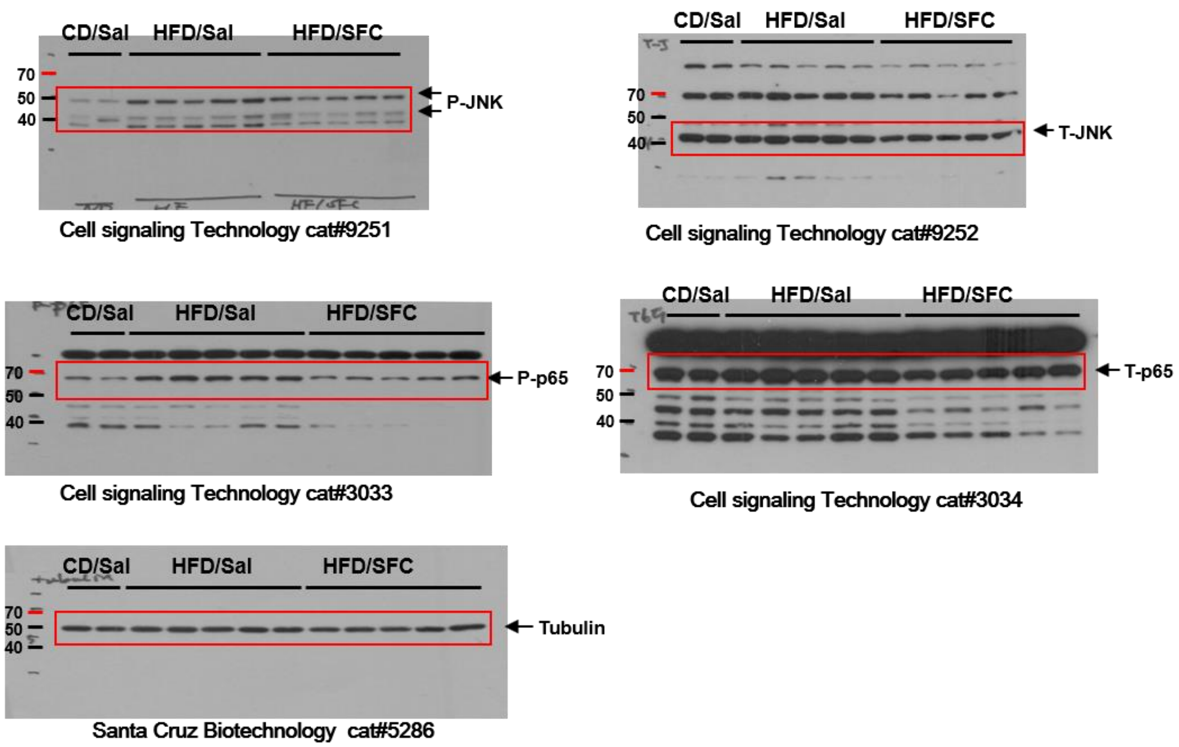

Whole blot images of western blotting analysis for P-JNK, T-JNK, P-p65, T-p65 and tubulin in Figure 4c. Cropped blots were designated by delineation with dividing lines. The molecular weights are indicated at left of ladders. Supplier and catalogue number of antibodies are also written below images.

Supplementary Figure 8

Full-length blots for cropped blots in Figure 4d

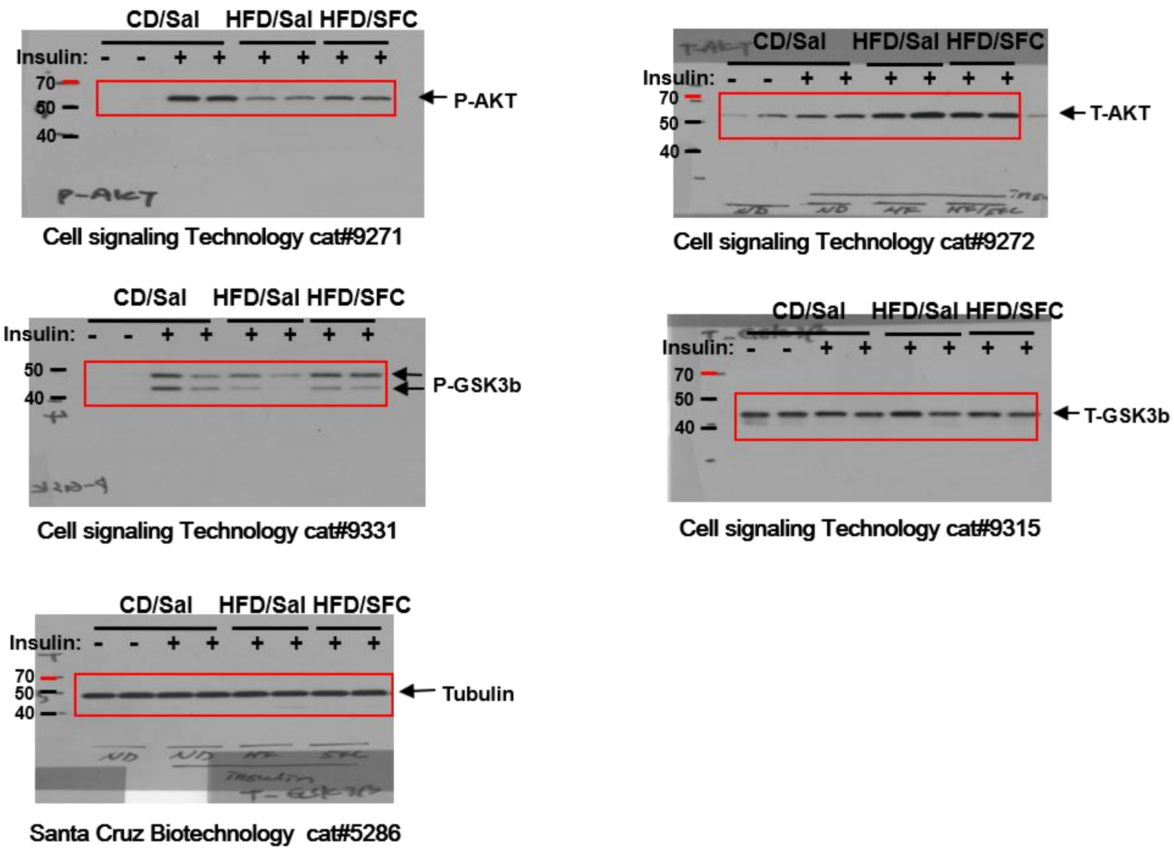

Whole blot images of western blotting analysis for P-AKT, T-AKT, P-GSK3b, T-GSK3b and tubulin in Figure 4d. Cropped blots were designated by delineation with dividing lines. The molecular weights are indicated at left of ladders. Supplier and catalogue number of antibodies are also written below images.
